# The American dream: Election night dreams predict overnight change in feelings about the election and the future

**DOI:** 10.1371/journal.pone.0357370

**Published:** 2026-09-03

**Authors:** William H. Livingston, Adam L. Putnam, Erin J. Wamsley

**Affiliations:** 1 Furman University Department of Psychology, Greenville, South Carolina, United States of America; 2 Furman University Department of Neuroscience, Greenville, South Carolina, United States of America; National Cheng Kung University, TAIWAN

## Abstract

Real-life experiences are often incorporated into our dreams. Does dreaming about those experiences affect how we feel about them? Prior studies have suggested that dreaming may attenuate emotional reactivity overnight, with participants feeling less negative about an experience after dreaming about it. The present study used the 2024 US presidential election to examine the relationship between dreaming and emotional reactivity in the context of a real-life emotional experience. We hypothesized that dreaming about the election would be associated with reduced negative feelings toward it the following morning. N = 931 American participants completed an online survey on the day of and the day after the 2024 US election. Participants rated their feelings about the election, their personal future, and the collective future of the country before and after election night and reported their dreams from that night. Contrary to our hypotheses, dreaming about the election was associated with *intensified* negative affect the next morning, including a more negative outlook on the personal and collective future. This pattern was specific to Democrats and Independents – dreaming about the election was not associated with overnight changes in affect among Republicans. Contrary to emotional regulation theories of dreaming, these observations suggest that sleep and dreaming are associated with amplified emotional reactivity, at least in the short-term.

Large-scale collective events can have dramatic effects on people’s dreams. After September 11^th^, 2001, Americans frequently dreamed of the attacks [1], and dream intensity was heightened [2]. Similarly, during the COVID-19 lockdowns, Americans frequently dreamed of the pandemic, and reported increases in dream intensity [3–5]. While the impact of national elections on dreams has not been previously studied, US presidential elections have been associated with sleep disruption in the population [6,7]. Do dreams following emotionally significant events serve a function? In this study, we leveraged a highly significant national event – the 2024 United States (US) presidential election – to examine how this collective experience affected dreams, and in turn, how those dreams predicted participants’ emotional appraisal of the election, as well as their feelings about their personal and collective future.

Dreaming has frequently been proposed to aid in overnight emotion regulation, especially during rapid eye movement sleep (REM), the sleep stage from which many of our most vivid dreams are recalled [8–12]. Evidence for this hypothesis comes, in part, from studies suggesting an emotion regulation function for sleep. Brain regions associated with emotion and emotion regulation are highly active during REM sleep, including the amygdala and medial-prefrontal cortex [11,13]. This activation corresponds with the highly emotional nature of REM dreams [14], and the fact that emotional experiences are preferentially incorporated into dreams [15]. Several studies have reported reduced reactivity to previously experienced emotional stimuli following sleep [16–18], although as described later, findings have been mixed [19].

There is also some evidence that dreaming, specifically, contributes to overnight emotion regulation. Mood is generally more positive in the morning, relative to the evening [20–22], and dreaming has been argued to contribute to this overnight shift. Early studies reported that overnight improvements in mood correlate with features of dream content [23], and that participants with depressed pre-sleep mood show a progressive positive shift in dream affect across the night [21]. Rosalind Cartwright’s classic studies of recently divorced persons support this in a clinical context, in that features of how participants dreamed of their ex-spouse predicted improvement in depression symptoms at follow-up [24,25]. A recent investigation appears to support this -- dreaming was associated with reduced next-morning emotional reactivity to previously-seen emotional stimuli, especially in persons with more positive dream affect [18]. Based on this and related evidence, REM sleep and dreaming have widely been proposed to *reduce* emotional reactivity to previously-experienced negative events, even as sleep *enhances* memory for those same experiences [10,12,26,27].

Dreaming about emotionally significant events may also affect how we think about the future. Humans frequently imagine possible futures [28], even dreaming about impending future-events during sleep [29,30]. These thoughts often center on our personal lives, such as considering what to have for dinner, worrying about an upcoming surgery, or planning a party for a loved one. But these thoughts can also relate to the future of the groups we are a part of, like when we worry about the outcome of a presidential election or imagine the selection of a new pope (termed *collective future thought (CFT)* [31]). Typically, Western samples display a positivity bias towards their personal future and a negativity bias towards the future of their country [32,33]. This may occur because people remember their personal past more positively than their collective history, due to how cultural life scripts and implicit trajectories guide future prospection and/or because current events differentially influence thoughts about the personal and collective future [32,34–36].

It is unclear how large-scale national events influence future thinking. Shrikanth et al. (2018) showed similar valence patterns in future thinking before and after the 2016 presidential election, suggesting minimal influence of that major national event. However, data collected during the height of the COVID-19 pandemic indicated that the personal positivity bias disappeared, but reappeared a year later [37], and that participants frequently mentioned the pandemic when thinking about the future, even without being instructed to do so [38]. Recent research on national elections held in 2024 indicated that phenomenological differences between collective remembering and collective future thinking are likely influenced by self-serving attitudes [39]. From a theoretical perspective, it seems that public events should shape future thinking. Research in autobiographical memory suggests unstable periods (e.g., during war) generate frequently remembered personal events [40–43]. Because dreaming has been associated with improved memory for events we dream about [44], and reduced emotional reactivity, dreams could potentially influence how large-scale collective events impact the valence of future thinking.

The current study investigated how the 2024 US presidential election – an event of great collective importance [45,46] – was represented in the dreams of Americans. We hypothesized that participants would be highly likely to dream about the election on the night of November 5th, 2024, owing to the emotional salience of the event. We also examined whether dreaming about the election was associated with changes in participants’ feelings toward it, as well as their feelings toward their own personal future and the future of the US. We expected that participants of any political orientation might experience negative feelings surrounding the election, and that dreaming about the election would be associated with an overnight reduction in these negative feelings. We also hypothesized that election-related dreams would be associated with an overnight shift toward a more positive outlook towards both the personal and collective futures.

## Method

### Overview and timing

Participants completed two surveys on consecutive days. The first survey opened at 8:00 AM ET on the day of the 2024 US presidential election between Republican candidate Donald Trump and Democratic candidate Kamala Harris (November 5th, 2024) with the last participant completing the survey at 11:55 AM ET, well before the first polls closed at 7:00 PM ET. This timing ensures that no participant knew any results at the time of the first survey. The second survey opened at 8:00 AM ET the following day, with the last participant completing the survey at 9:54 AM ET. While Donald Trump’s election victory was announced by the Associated Press at 5:35 AM ET on November 6th, earlier calls had been made by other news outlets (notably, FOX News called the race at 1:47 AM ET). Results from key states were known even earlier – for example, Trump was already strongly leading by 1:24am EST, when the AP announced he had won Pennsylvania [47]. But critically, no participants knew the outcome of the election while completing the first survey and all participants had learned of the outcome by the time they completed the morning follow-up survey.

### Participants

A convenience sample of people currently residing in the United States over the age of 18 was recruited via Prolific (www.prolific.com). Given that the effect size of interest was unknown, our goal was to recruit as many participants as possible given available funding. *N* = 1000 participants enrolled in the study and completed the first survey on the day of the US presidential election, and *N* = 937 participants also completed the 2^nd^ survey the day after the election. We excluded six participants for either not reporting personal or collective future ratings on the first survey or for having a dream report of less than 5 words, leaving a final sample size of *N* = 931 (mean age = 41.96 ± 12.58 *SD*; 434 male / 494 female / 3 Other). Participants received $2.50 for completing both surveys. This research was approved by the Furman University IRB Committee and all participants provided written consent to participate. Due to the time-sensitive nature of this project, this study was not pre-registered.

The political orientation of the final sample is described in Table 1. While political views are not an immutable trait, for the sake of convenience, people who self-identified as members of a specific party will hereafter be referred to by the name of that party (i.e., people who identified as liberal, average, or moderate Democrats will be referred to as “Democrats”, people who identified at Independents will be referred to as “Independents”, and people who identified as conservative, average or moderate Republicans will be referred to as “Republicans”). There were no significant differences in average age between Republicans (M = 42.43, SD = 12.79), Independents (M = 39.92, SD = 11.40), and Democrats (M = 42.46, SD = 12.83), F(2,928) = 2.87, p = .057, η²ₚ = .006.

**Table 1:** Participant Counts by Political Orientation.

Political Orientation	Total N	# Recalled Dream	# Dreamed about Election
Republican	397 (42.64%)	290 (31.15%)	137 (14.72%)
Moderate	121 (13.00%)	82 (8.81%)	33 (3.54%)
Average	79 (8.49%)	55 (5.91%)	24 (2.58%)
Conservative	197 (21.16%)	153 (16.43%)	80 (8.59%)
Independent	176 (18.90%)	124 (13.32%)	45 (4.83%)
Democrat	358 (38.46%)	243 (26.10%)	142 (15.25%)
Moderate	106 (11.39%)	70 (7.52%)	37 (3.97%)
Average	105 (11.28%)	68 (7.30%)	34 (3.65%)
Liberal	147 (15.79%)	105 (11.28%)	71 (7.63%)
Total	931	657 (70.57%)	324 (34.80%)

*Notes*. Total N = all participants who completed both surveys. # Recalled Dream = participants who reported having dreamt. # Dreamed about Election = participants who reported having dreamt specifically about the election. Percentages are out of all N = 931 participants who completed both surveys.

### Procedures

We administered the surveys via Qualtrics (Qualtrics, Provo, UT). During the first survey on the day of the presidential election, participants reported demographic information, including self-reported political identity, with the options “Liberal Democrat”, “Average Democrat”, “Moderate Democrat”, “Independent”, “Moderate Republican”, “Average Republican”, and “Conservative Republican”.

After this, participants completed the Positive and Negative Affect Schedule (to provide a snapshot of their current mood; PANAS [48]). They then rated their feelings towards the election (“*How are you feeling about the election and the events surrounding it?*”) using a 10-point Likert scale, as well as their own personal future (“*Overall, how do you feel about the future of your personal life?*”) and the future of the US (“*Overall, how do you feel about the future of the United States of America?*”) using 100-point Likert scales, with all scales ranging from “Extremely NEGATIVE” to “Extremely POSITIVE”. At the end of the first survey, we told participants: “…*please try and remember your dreams tonight, as you will be asked to report them on tomorrow morning’s survey*”. Additionally, participants were instructed to complete the second survey as soon as possible after waking up the next morning.

The day after the election, participants completed a second survey in which they indicated whether they recalled a dream from the previous night. If so, they were prompted to report everything they could remember about the dream, using the following instruction adapted from the Most Recent Dream report collection method [49]:

*Below, please tell us everything you remember dreaming last night. Please describe the dream exactly and as fully as you remember it. Your report should contain, whenever possible: a description of the setting of the dream, whether it was familiar to you or not; a description of the people, their age, gender, and relationship to you. If possible, describe your feelings during the dream and whether it was pleasant or unpleasant. Be sure to tell exactly what happened during the dream to you and the other characters. Even if the dream is just a simple, fleeting thought or image, please describe it in as much detail as you can.*

Participants then reported whether their dream was related to the current election (*yes/no*), and if so, they described how it was related. They also were asked whether the dream was related to any other “*specific past experience*” that “*happened at a particular place and time*”, and if so, they described this experience. If they believed their dream was related to multiple other past experiences, participants were instructed to focus on the one they were “*most certain*” related to the dream.

Participants then indicated whether they voted, and if they knew who won the election (all participants correctly stated that Donald Trump won the election). Finally, participants again completed the same ratings from the first survey about their feelings towards the election, their personal future, and the future of the United States, as well as the PANAS.

### Dream scoring

For analysis, we used participants’ own subjective response to quantify whether their dream was related to the election. Reports were further scored for election-related content by the large language model *gpt 4o-mini* from OpenAI (openai.com), selectively for the purpose of rating how directly/strongly the election was represented in each dream, and to assess whether any other past experiences participants cited as a dream source also related to elections. To validate *gpt 4o-mini’s* scores, a subset of 180 reports were also scored by 2 human raters. *Gpt 4o-mini* scoring was conducted in R via OpenAI’s API, using the *chatgpt* package [50]. Along with a binary decision about whether the report contained any content, dreams were classified according to whether they were *directly*-related to the 2024 election, related to *other elections*, *indirectly*-related to elections, or *unrelated* to elections. Any other past experiences that participants listed as a source of their dream were also classified according to the presence of election-related content. A more complete description of the scoring methodology is in the supplemental materials (S1 Supplementary Information). The full set of scoring instructions provided to raters is available on Open Science Framework at https://osf.io/69qwu/.

*Gpt 4o-mini* agreed with the human raters on whether reports contained content in an average of 97.8% of cases (compared to 98.9% agreement between the two human raters). *Gpt 4o-mini* and the human raters had 81.0% agreement on whether dreams were related to the election (compared to 88.8% agreement between the two human raters), and 75.0% agreement on whether any other past experiences participants cited as a dream source also related to elections (compared to 95.2% agreement between the two human raters). Human raters and *gpt 4o-mini* had an average of .80 reliability for how *directly* dreams related to the election (Spearman’s rho, as compared to .86 agreement between human raters).

Thus, for the most part, *Gpt 4o-mini* was reliable with human raters, and its reliability with humans approached human-to-human reliability. The exception was when *Gpt 4o-mini* was asked whether *other* past episodic memories indicated as a dream source were also related to elections. The reasons why *Gpt 4o-mini* struggled with this particular decision are unclear, as the instructions were seemingly straightforward. The results of this rating, which form a very small part of the reported results, should be taken with caution.

### Statistical analyses

The primary dependent measures were overnight change in feelings about the election, overnight change in feelings about the personal future, and overnight change in feelings about the collective future. All overnight change scores were computed by subtracting the rating given on the day before the election from the rating given on the day after the election. Statistical analyses were conducted in R (R Core Team, 2021), and used the *rstatix* package (Kassambara, 2019). For simplicity, in the analyses below, political orientation is collapsed into the 3 superordinate categories of Democrat (“Liberal Democrat”, “Average Democrat” or “Moderate Democrat”), Independent, and Republican (“Moderate Republican”, “Average Republican”, or “Conservative Republican”). Parallel analyses utilizing all 7 political categories are reported in the Supplementary Results (S1 Supplementary Information), which yield largely identical results. Additionally, pre-election baseline analyses are reported in the Supplementary Results (S1 Supplementary Information). Critically, those who did and did not dream about the election did not differ significantly in pre-election feelings towards the election, their personal future, or the collective future (all *p* ≥ .538). Data and analysis code are available in the Open Science Framework repository: https://osf.io/69qwu/.

## Results

### Representation of the election in dreams

657 participants reported a dream (70.57% of all participants). Of those, 324 (49.32%) said that their dream was related to the 2024 US presidential Election (Table 1). This was closely aligned with *gpt 4o-mini’s* ratings, in which 286 (43.53%) reports were classified as related to the election. When scored for election-relatedness by *gpt 4o-mini,* 153 (47.22%) election-related dreams were classified as directly related to the 2024 US presidential election, 55 (16.98%) as related to elections, but not specifically the 2024 US presidential election, and 74 (22.84%) as only indirectly related to elections. Table 2 lists examples of election-related dreams.

**Table 2:** Examples of Dream Reports.

Election Incorporation	Dream Report	Rating
Direct Incorporation of 2024 Election	“I had a dream where I was watching the television in bed by myself and Harris was elected president. On the tv it was her walking across a stage that had us flags on it and she was waving. The text in the bottom said that she had won…”	3
Direct Incorporation of Other Election Content	“It was at night on a dark street and poll stations were all over. I was walking and no one was at the polls. I tried to vote and I couldn’t get help the machines weren’t working. I was upset and it was cold out. I started to become fearful like something happened.”	2
Indirect Incorporation	“I remember being in a large room with lots of people that I didn’t know. We were all trying to get into some sort of a line, but they were different lines. The lines weren’t for anything important... I remember feeling, confused and a little nervous about trying to figure out which line I needed to get into. There was lots of talking in the dream but they weren’t talking to me and I wasn’t talking to them. I remember thinking to myself ‘where am I supposed to go’…”	1
No Incorporation	“I was in school, but I couldn’t find my locker and I didn’t know what classes to go to. After that, I realized that I missed every class that semester because I didn’t know that I was enrolled.”	0

*Notes*. Examples of dream reports given by participants on the post-election survey, which occurred the morning after the presidential election (November 6^th^, 2024).

Participants reported that 275 dreams (41.86%) were also related to another specific past experience of theirs, besides the 2024 US presidential election. Other past sources were least common for dreams directly related to the 2024 election (3.57%), and increasingly common for dreams related to other elections (4.37%), indirectly related to elections (26.98%), or unrelated to the election (65.08%). Of these other past experiences, 88 (32.00%) were also related to elections.

### Dream recall

Whether participants recalled a dream or not was unrelated to overnight change in feelings about the election (*t*(523) = 0.09, *p* = .928, *d* = 0.01), their personal future (*t*(515) = 0.54, *p* = .59, *d* = 0.04), or the future of the country (*t*(497) = 0.69, *p* = .489, *d* = 0.05). The remaining analyses of dream features below consider only the 657 participants who successfully recalled and reported a dream.

### Overnight change in feelings about the election

**Fig 1:**
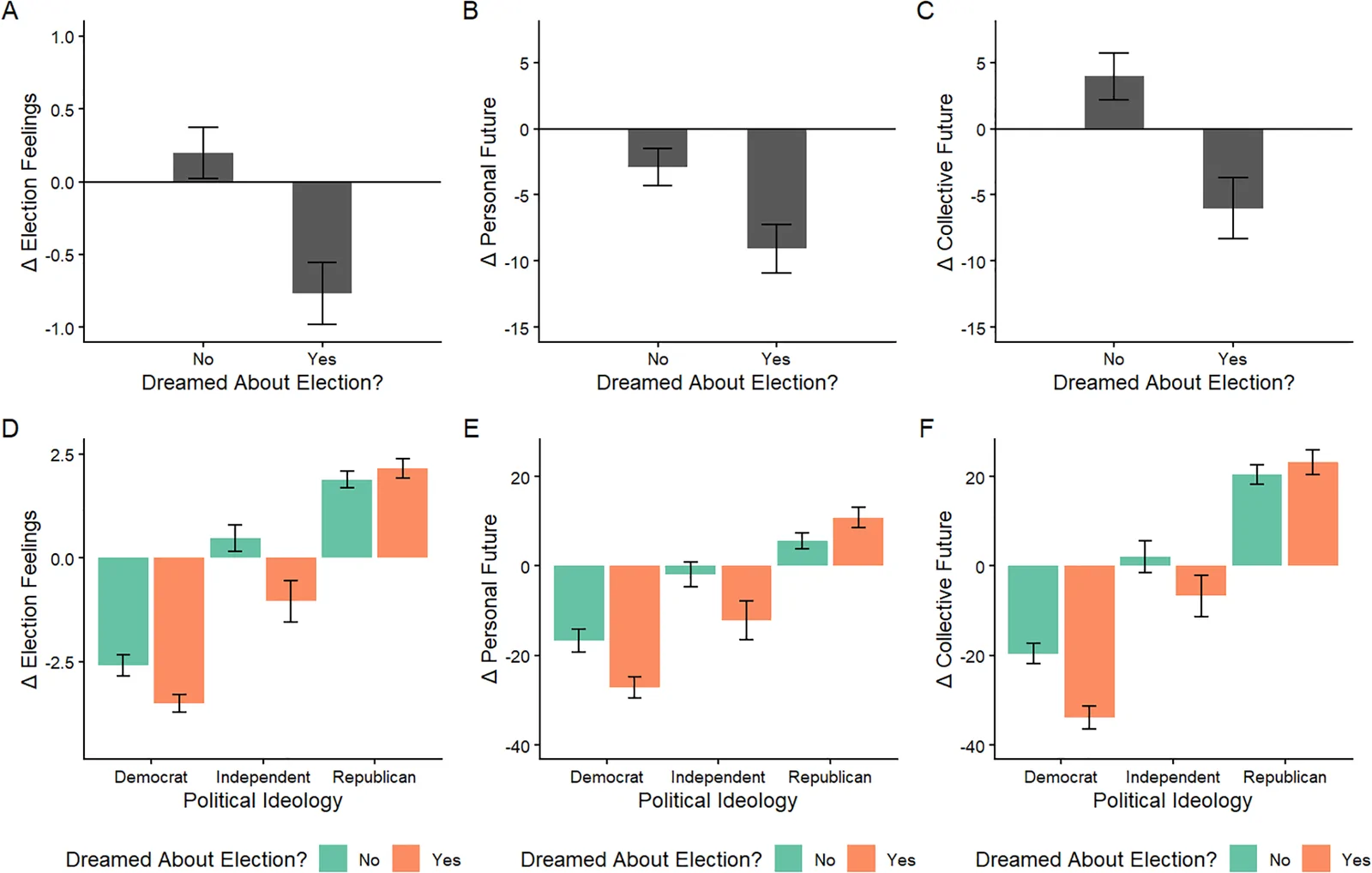
Dreaming and Overnight Change in Feelings About the Election and the Future. **A-C)** Participants who dreamed about the election showed a larger negative shift in attitudes toward the election, their personal future, and the collective future. Error bars = ± SEM. **D-F)** This effect depended on political orientation, with Democrats and Independents feeling more negatively after dreaming about the election, but Republicans feeling more positively. Error bars = ± SEM.

We examined how dreaming about the election was associated with overnight change in feelings toward it using a 2 (election dream: yes/no) x 3 (political orientation: Democrat/Independent/Republican) ANOVA, with overnight election affect change as the dependent variable. There was a main effect of political orientation (*F*(2, 651) = 229.50, *p* < .001, η²ₚ = .414; Table 3), such that Democrats showed a significant overnight shift toward more negative affect, Independents showed a small overnight shift toward more negative affect, and Republicans showed a significant overnight shift toward more positive affect (all pairwise differences *p* < .001). There was also a main effect of dreaming about the election (*F*(1, 651) = 9.90, *p* = .002, η²ₚ = .015; Fig 1A). Contrary to our prediction, people who dreamed about the election showed a stronger *negative* shift in their feelings toward the election overnight, compared to those who did not dream about the election. Critically, there was also an election dream x political orientation interaction (*F*(2, 651) = 5.75, *p* = .003, η²ₚ = .017). Among Democrats and Independents, dreaming about the election was associated with feeling more negatively about the election the next day (Democrats: *t*(215) = 2.72, *p* = .007, *d* = 0.35; Independents: *t*(79) = 2.56, *p* = .012, *d* = 0.49; Fig 1D). In contrast, Republicans felt similarly about the election regardless of whether they dreamed about it (*t*(272) = 0.87, *p* = .385, *d* = 0.10). In other words, dreaming about the election was associated with a stronger overnight shift in affect toward it for Democrats and Independents.

**Table 3:** Overnight Affect Change by Political Orientation.

Participant Classification	Political Orientation	Election Affect		Personal Future		Collective Future	
Mean	±SD	Mean	±SD	Mean	±SD
All (N = 931)	Republican	2.05	±2.61	8.15	±23.63	22.69	±30.08
	Independent	0.02	±3.09	−4.46	±24.98	−0.43	±31.09
	Democrat	−3.03	±2.59	−23.18	±28.77	−28.89	±27.77
Recalled Dream (N = 657)	Republican	2.01	±2.63	8.06	±24.69	21.73	±29.90
	Independent	−0.07	±3.11	−5.64	±26.42	−1.09	±31.67
	Democrat	−3.12	±2.62	−22.81	±27.81	−27.97	±28.37
Dreamed about Election (N = 324)	Republican	2.15	±2.81	10.76	±26.68	23.14	±32.64
	Independent	−1.04	±3.38	−12.18	±29.43	−6.67	±31.09
	Democrat	−3.50	±2.58	−27.19	±28.21	−33.92	±30.14

*Note*. Values represent the change in rating from the day before to the day after the election. Negative numbers indicate an overnight shift toward more negative affect, and positive numbers indicate an overnight shift toward more positive affect. All = all participants who completed both surveys. Recalled Dream = participants who reported having dreamt. Dreamed about Election = participants who reported having dreamt specifically about the election, in any manner.

### Dreaming and future thinking

We next examined how dreaming about the election was associated with overnight changes in feelings about the personal and collective future, using two 2 (election dream: yes/no) x 3 (political orientation: Democrat/Independent/Republican) ANOVAs. There were significant main effects of dreaming about the election; those who dreamed about the election showed a negative shift in feelings about the future across the night, compared to those who did not dream about the election (personal future: *F*(1, 651) = 5.56, *p* = .019, η²ₚ = .008, Fig 1B; collective future: *F*(1, 651) = 7.35, *p* = .007, η²ₚ = .011, Fig 1C).

Again, there was an election dream x political orientation interaction, in that the effects of dreaming about the election depended on political orientation for both the personal (*F*(2, 651) = 7.13, *p* < .001, η²ₚ = .021, Fig 1E) and collective future (*F*(2, 651) = 5.63, *p* = .004, η²ₚ = .017, Fig 1F). Democrats who dreamed about the election felt more negatively toward their personal (*t*(225) = 2.99, *p* = .003, *d* = 0.39) and collec*t*ive future (*t*(239) = 4.17, *p* < .001, *d* = 0.53) the next day, compared to Democrats who did not dream about the election. Independents who dreamed about the election also felt more negatively toward their personal future the next day, compared to Independents who did not dream about the election (*t*(77) = 1.99, *p* = .050, *d* = 0.38); However, dreaming about the election did not significantly predict changes in feelings towards the collective future among Independents (*t*(93) = 1.50, *p* = .138, *d* = 0.28). Finally, for Republicans, dreaming about the election was not significantly associated with overnight change in feelings about either the personal (*t*(268) = 1.75, *p* = .081, *d* = 0.21) or collective fu*t*ure (*t*(266) = 0.75, *p* = .453, *d* = 0.09). Thus, again, dreaming about the election was associated with a stronger overnight shift in feelings about the future for Democrats and Independents.

### Form of election incorporation into dreams

**Fig 2:**
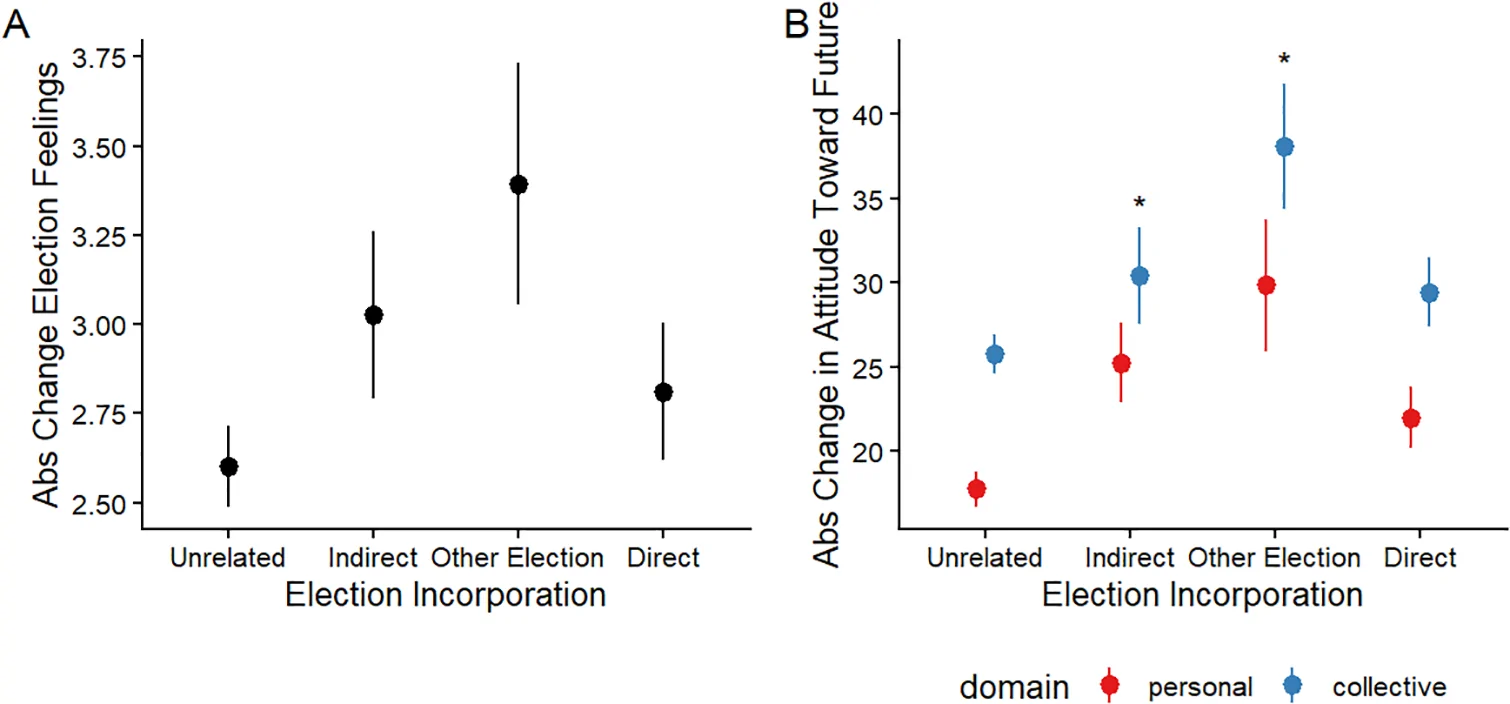
Form of Election Incorporation into Dreams and Overnight Change in Feelings. **A)** Change in feelings about the election across the night as a function of the form in which the election appeared in dreams. No two incorporation categories differed significantly from each other after Holm correction for multiple comparisons Y-axis = absolute value of overnight change in feelings about the election. Error bars = ± SEM. **B)** Change in feelings about the personal and collective future across the night as a function of the form in which the election appeared in dreams. * = differs significantly from “Unrelated” category after Holm correction for multiple comparisons. Y-axis = absolute value of overnight change in feelings about the election. Error bars = ± SEM.

Next, we examined whether how *directly* the election appeared in dreams predicted overnight change in participants’ feelings, based on *gpt 4o-mini’s* ratings of election incorporation (see Methods). While there was a near-significant association between how directly the election appeared in dreams and overnight change in feelings about the election (*F*(3, 653) = 2.47, *p* = .061, η²ₚ = .011), no two categories of incorporation strength differed significantly from each other after correction for multiple comparisons (Fig 2A). However, how directly participants incorporated the election into their dreams was associated with how their feelings changed overnight about their personal future (one-way ANOVA: *F*(3, 653) = 6.86, *p* < .001, η²ₚ = .031, Fig 2B) and the future of the country (one-way ANOVA: *F*(3, 653) = 4.83, *p* = .002, η²ₚ = .022). Specifically, dreams indirectly related to elections (*t*(106) = 2.87, *p* = .025, *d* = 0.36) or related to other elections (*t*(63) = 2.99, *p* = .024, *d* = 0.48) were associated with greater overnight change in feelings toward the personal future, relative to election-unrelated dreams (Fig 2B). But notably, dreams directly mentioning the 2024 Presidential election did not differ significantly from election-unrelated dreams (*t*(264) = 2.04, *p* = .171, *d* = 0.20). The pattern for feelings toward the collective future was similar, but only dreams about other elections were significantly associated with overnight change (*t*(66) = 3.16, *p* = .014, *d* = 0.49). Together, these data suggest that dreaming about the election was associated with stronger overnight change in feelings about the future when they represented the election in an indirect manner.

### Mood as a predictor of election dreams (PANAS)

Mood on the day of the election was analyzed using a 2 (yes/no election dream) x 3 (Democrat/Independent/Republican) ANOVA, with pre-election PANAS scores as the dependent variable. There was a main effect of dreaming about the election, such that negative affect was stronger in participants who later dreamed about the election, compared to those who did not (*F*(1, 651) = 7.38, *p* = .007, η²ₚ = .011). In contrast, this effect failed to reach significance for positive affect, *F*(1, 651) = 3.57, *p* = .059, η²ₚ = .005. In both cases, the association between evening affect and election dreams was unrelated to political orientation (no political orientation x election dream interaction for positive affect: *F*(2, 651) = 0.79, *p* = .456, η²ₚ = .002; for negative affect: *F*(2, 651) = 1.73, *p* = .178, η²ₚ = .005).

We examined changes in mood across the night using 2 (election dream: yes/no) x 3 (political orientation: Democrat/Independent/Republican) ANOVAs. There were significant main effects of dreaming about the election, with those who dreamed about it showing a greater increase in negative affect across the night, as well as a greater decrease in positive affect, compared to those who did not dream about it (negative: *F*(1, 651) = 16.53, *p* < .001, η²ₚ = .025; positive: *F*(1, 651) = 4.52, *p* = .034, η²ₚ = .007).

Here again, the association between dreaming and overnight change in affect depended on political orientation (election dream x political orientation interaction for negative affect: *F*(2, 651) = 6.43, *p* = .002, η²ₚ = .019; for positive affect: *F*(2, 651) = 5.51, *p* = .004, η²ₚ = .017). Among Democrats, dreaming about the election was associated with increased negative affect (*t*(205) = 3.46, *p* < .001, *d* = 0.45) and decreased positive affect the next day (*t*(239) = 3.36, *p* < .001, *d* = 0.43). Among Independents, dreaming about the election was also associated with increased negative affect the next day (*t*(62) = 2.72, *p* = .008, *d* = 0.54), but the effect for positive affect did not reach significance (*t*(94) = 1.33, *p* = .188 *d* = 0.25). In contrast, for Republicans, dreaming about the election was not significantly associated with overnight change in either negative (*t*(219) = 0.37, *p* = .715, *d* = 0.04) or positive affect (*t*(260) = 1.16, *p* = .247, *d* = 0.14). Thus, dreaming about *t*he election was again associated with stronger affect changes in Democrats and Independents.

### Changes in personal vs. collective future thinking over time

**Fig 3:**
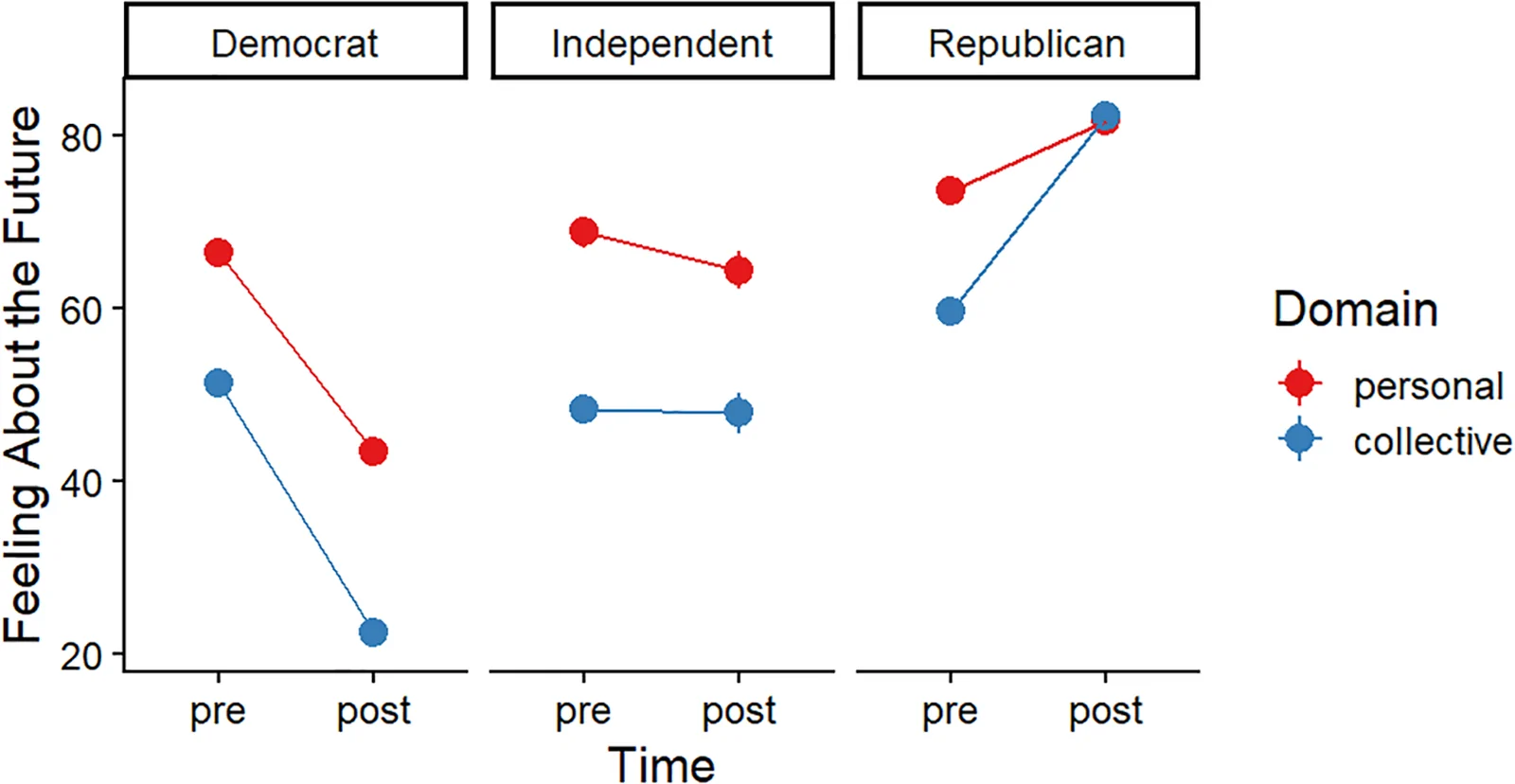
Feelings About Personal and Collective Future Converge Overnight in Republicans.

To assess how feelings toward the personal and collective future changed across the night, we ran a 2 (time: before/after election) x 2 (domain: personal/collective) x 3 (political orientation: Democrat/Independent/Republican) ANOVA. Overall, participants felt more positive about their personal future than about the future of the United States (*F*(1, 928) = 461.38, *p* < .001, η²ₚ = .332, Fig 3). But this was qualified by a three-way interaction between time, domain, and political orientation, indicating that political orientation predicted changes in how people felt about the future across the night (*F*(2, 928) = 46.61, *p* < .001, η²ₚ = .091, Fig 3). Among Democrats, feelings about the future became more negative across the night (main effect of time: *F*(1, 242) = 274.68, *p* < .001, η²ₚ = .532), somewhat more so in the personal than collective domain (time x domain interaction: *F*(1, 242) = 7.37, *p* = .007, η²ₚ = .030, Fig 3B). Among Independents, there was no change in feelings about the future (no main effect of time: *F*(1, 123) = 2.00, *p* = .159, η²ₚ = .016). Among Republicans, feelings about the future became more *positive* across the night (*F*(1, 289) = 111.54, *p* < .001, η²ₚ = .278), and much more so in the collective than the personal domain (*F*(1, 289) = 77.50, *p* < .001, η²ₚ = .211, Fig 3B). As a result, Republicans’ feelings about the personal and collective future converged on the day after the election, becoming indistinguishable from each other (*t*(289) = 0.90, *p* = .367, *d* = 0.05).

For Democrats and Independents, feelings about the personal and collective future changed in a similar manner overnight. Selectively for Republicans, feelings about the collective future increased more over time than did feelings about the personal future, leading to a convergence in participants’ assessment of their personal and collective futures. Error bars = ± SEM.

## Discussion

Important collective events influence people’s dreams. We asked how dreaming about the 2024 US presidential election might predict overnight changes in feelings toward it. Based on prior evidence that dreaming is associated with reduced emotional reactivity, we hypothesized that those who dreamed about the election would feel less negative about it the following morning. But our observations demonstrate just the opposite -- dreaming about the election was associated with an *increase* in emotional intensity across the night. Democrats’ feelings about the election became more negative across the night, and this shift was greater among those who dreamed about it. This was similar for Independents, who also saw feelings about the election become more negative across the night among those who dreamed about it. In contrast, Republicans’ feelings about the election became more positive across the night, but this shift was not associated with dreaming about the election. Analogous patterns were observed for how participants felt about their own future and the future of the United States. Although election dreams were only weakly associated with overnight affective changes in the full sample, when political orientation was taken into account the observed effects sizes were substantial (Cohen’s d ranged up to ≈0.5 for within-political group comparisons).

For decades, dreaming has been hypothesized to play a role in “emotional adaptation” to stressful events, reducing emotional reactivity across the night [10,21,23]. But empirical evidence for this hypothesis has always been limited. While correlational, our current observations could signal that dreaming about an event could be associated with *enhanced*, rather than attenuated, emotional reactivity towards that event. There are prior reports of enhanced emotional reactivity following sleep. In several studies, sleeping after encoding negative images amplified negative emotional reactions to them [51,52]. Likewise, there is analogous evidence for a specific association between dreaming and enhanced next-morning emotional reactivity. In one study, participants watched a stress-inducing movie; the next morning, people who reported dreaming about the movie showed a stronger emotional response [53]. In another study, REM-deprived participants showed *greater* attenuation of reactivity to negative images, and next-morning emotional reactivity was *positively* associated with having experienced emotionally intense dreaming [54]. These observations are congruent with the current study in suggesting that sleeping after an emotional event and dreaming about it is associated with enhanced rather than attenuated emotional reactivity toward it.

Our results also have implications for understanding collective future thinking. Our election night data mirrored the typical pattern seen in past research: people felt more optimistic about their personal future than the future of their country, regardless of political orientation (Fig 3). This pattern carried over to the post-election survey for both Democrats and Independents. But on the morning after the election, Republicans felt equally positive about their personal and collective futures. To our knowledge, this is the first time a Western sample has displayed such a similar outlook towards their personal future and the future of their country their country, although this is consistent with other research examining future thinking and memory in relation to winning elections [39,55]. This convergence between the personal and collective in Republican participants may indicate “basking in reflected glory” [56] – people feel good about their group when their group does well – or could indicate that Republicans felt closer to their country following the election. Prior research has demonstrated that participants are more optimistic about the future of groups they have a stronger relation to, relative to groups that are less central, such as family versus country [57]. We suspect that Republicans viewed the country as much more related to themselves following Donald Trump’s victory, leading to the drastic increase in CF ratings following the election. This relates closely to the idea of “public mood”, a phenomenon in which the general mood of the public is influenced by the groups of which they are members [58,59]. In line with this concept, regardless of dreaming, Democrats overwhelmingly felt worse in the morning towards the election, their personal future, and the future of the country, while Republicans felt overwhelmingly better.

Of particular note was the high rate of dream recall amongst our participants, with over 70% reporting a dream from the night of the election, and nearly half of those dreams relating to the election. While we expected participants to dream about the election frequently due to the salience of the experience, other factors may have influenced these high rates of recall as well. First, it is possible our explicit instruction to remember dreams may have increased recall. It is also possible that sleep disruption caused by the election may have amplified dream recall. Elections have been demonstrated to influence sleep, including by decreasing the total amount of sleep obtained, increasing WASO (wake after sleep onset), and decreasing sleep efficiency [6,7]. Although a limitation of this study is that sleep data were not collected, it is possible that participants’ sleep was disrupted, which in turn may have increased their likelihood of remembering a dream simply by increasing the number of opportunities they had to awaken and encode dream experience to memory [60,61]. Elevated dream recall may also help to explain the high number of election-related dreams reported in this study. Dream content is notoriously difficult to predict, and this is a very high rate of event incorporation relative to what is typically seen in laboratory studies [62]. Election-related dreams were also remarkable in the extent to which they very directly and transparently represented the event – 23% of all dreams specifically mentioned the candidates and/or other imagery associated with this particular election. This is uncommon in laboratory studies, where dreams participants perceive to be “about” a specific past event rarely resemble that event with any fidelity [63].

But contrary to our hypotheses, dreams *indirectly* related to the election most strongly predicted changes in how participants thought about the future. This could be because these dreams integrated memories of the election with other past experiences. Indeed, participants were more likely to indicate a second episodic memory source for dreams that were indirectly related to the election, as compared to those that were directly related to the election. There is strong evidence that dreams are often constructed from multiple past memories, with dreams being *triggered by* a specific recent experience, but not resembling any particular past event very strongly [29,30,64]. Rosalind Cartwright’s work with recent divorcees supports the hypothesis that dreams about emotional experiences affect next-day emotions more strongly when they integrate other memory sources — participants felt better about their situation after dreaming about their former spouse, but *only when the dream drew from other memory sources as well* [24]. This could explain why indirect incorporation was associated with increased affect change overnight, but direct incorporation was not – when an emotional experience is more integrated with our other memories and experiences, its reactivation during sleep may be more likely to affect our appraisal of the future.

### Limitations

Of course, as we did not experimentally manipulate dreaming in the current study, we cannot make confident conclusions about the causal contribution of dreams to overnight changes in participants’ feelings. Although these data are consistent with a causal role for dreaming in affective processing, a variety of third variables might instead explain the association between election dreams and overnight intensification of participants’ feelings. It is possible, for example, that greater investment in the election could have increased the likelihood of dreaming about it, while independently affecting participants’ feelings towards the election. Another possibility is that viewing more election coverage before sleep could both increase the likelihood of election dreams and intensify overnight change in participants’ feelings toward the election. Participants who dreamed about the election could indeed have differed from those who did not on any number of unmeasured variables, such as stress sensitivity, political engagement, or sleep quality.

Another important limitation of this study is that we examined dreams on only a single night, during which the emotional event of the presidential election was ongoing. If sleep and dreaming do serve an emotional adaptation function, this could be a process that unfolds across multiple nights. Future research could examine how dreaming about an emotional experience is associated with changes in feelings toward that experience across longer times spans (such as months or years).

Finally, it is important to note the limitations of using a single question to assess political orientation. Political identity is complex and multidimensional in ways that are not captured here. It is possible that, for instance, a person could identify on the left side of the political spectrum but not as a Democrat – on the scale we used here, political party and political ideology are conflated. Future studies should consider separately assessing party affiliation and ideology across multiple questions.

## Conclusion

Here, we investigated how a highly emotional collective experience was incorporated into dreams. Nearly half of participants who recalled a dream dreamed about the election. Contrary to our expectations, dreaming about the election was associated with intensified emotions toward it, toward the personal future, and toward the collective future. These observations contradict the longstanding hypothesis that dreaming functions to *reduce* emotional reactivity following an emotional event, instead suggesting that dreaming about an experience is associated with *greater* reactivity.

## Supporting information

S1 Supplementary Information Supplementary Methods and Results. (DOCX)
