# Supplementary material for: The American dream: Election night dreams predict overnight change in feelings about the election and the future
Source: PLoS One. 2026 Sep 3;21(9):e0357370. doi: 10.1371/journal.pone.0357370 (PMC13541154; doi:10.1371/journal.pone.0357370)
Supplement: Supplementary file 1 [file pone.0357370.s001.docx]

**Supplemental Materials**

**The American Dream:**

**Election Night Dreams Predict Overnight Change in Feelings about the Election and the Future**

William H. Livingston, Adam L. Putnam & Erin J. Wamsley

**Supplemental Methods**

**Dream Scoring by Raters**

Both the human raters and *gpt 4o-mini* scored each dream report on 3 dimensions. First, raters made a binary decision as to whether the report contained any content describing a mental experience prior to awakening (as opposed to stating, e.g., “*I don’t know*” or “*I don’t remember*”). Reports that did contain content were then scored on how strongly they related to the 2024 US presidential election. Reports were classified as either:

a) *Directly related*. The report incorporated content that unambiguously and specifically referred to the 2024 US Presidential election, for example by directly mentioning the current election or the candidates.
b) *Related to other elections*. The report incorporated content unambiguously related to elections, but not necessarily to the 2024 US Presidential election, specifically. This might include elements such as campaign signs, voting booths, ballots, voting, politicians, or other objects, actions, or characters that are specifically related to elections.
c) *Indirectly related.* The report incorporated content only *indirectly* related to elections. This might include themes about competitions, contests, debates, politics, newscasts, crowds, or leadership, but which are not specifically related to elections.
d) *Unrelated.* The report did not contain any content related to elections.

Any other past experiences that participants listed as a source of their dream were also classified according to the presence of election-related content. These rating procedures mirror those used in our earlier research on the incorporation of waking events into dreams [1–3]. The full set of scoring instructions provided to raters is available on Open Science Framework at <https://osf.io/69qwu/>.

**Statistical Analysis**

***Strength of Incorporation Analyses***

We used one-way ANOVAs to examine the relationship between how directly the election appeared in dreams and the absolute magnitude of overnight change in feelings about the election, the personal future, and the collective future. In these analyses, strength of incorporation was treated as a categorical independent variable. Here, we chose *not* to examine interactions with political orientation, due to the very large number of subdivisions it would create. We accounted for the fact that overnight change in our main dependent measures was in the opposite direction for Democrats and Republicans (as shown in Figure 1 in the main text) by using the absolute value of rating change as the outcome measure in these analyses. Wherever multiple pairwise comparisons were conducted, Type I error was controlled using the Holm correction.

1.	Wamsley EJ, Perry K, Djonlagic I, Reaven LB, Stickgold R. Cognitive replay of visuomotor learning at sleep onset: temporal dynamics and relationship to task performance. Sleep. 2010;33: 59–68.

2.	Wamsley EJ, Trost T, Tucker M. Memory updating in dreams. Sleep Adv. 2024;5: zpae096. doi:10.1093/sleepadvances/zpae096

3.	Wamsley EJ, Stickgold R. Dreaming of a learning task is associated with enhanced memory consolidation: Replication in an overnight sleep study. Journal of Sleep Research. 2018 [cited 25 Aug 2018]. Available: https://onlinelibrary.wiley.com/doi/abs/10.1111/jsr.12749

**Supplemental Results**

**Pre-Election Baseline**

Before the election, feelings about it did not differ between those who later dreamed about it, and those who did not (*F*(1, 651) = 0.33, *p* = .567, η²ₚ = <.001). This did not depend on political orientation (political orientation x election dream interaction: *F*(2, 651) = 0.52, *p* = .594, η²ₚ = .002). Similarly, before the election, feelings about the future did not differ between those who later dreamed about the election, and those who did not (personal future: *F*(1, 651) = 0.38, *p* = .538, η²ₚ < .001; collective future: *F*(1, 651) = 0.003, *p* = .954, η²ₚ < .001). This also did not depend on political orientation (political orientation x election dream interaction for personal future: *F*(2, 651) = 0.505, *p* = .604, η²ₚ = .002; for collective future *F*(2, 651) = 0.549, *p* = .578, η²ₚ = .002).

**Table S1**

*Baseline Affect Ratings by Collapsed Political Orientation.*

| Affect Measurement | Democrat | | Independent | | Republican | | *p-value* |
| --- | --- | --- | --- | --- | --- | --- | --- |
|  | Mean | ±SD | Mean | ±SD | Mean | ±SD |  |
| PANAS NA | 18.37^B,C^ | 7.69 | 15.80^A^ | 7.56 | 15.94^A^ | 6.87 | ** |
| PANAS PA | 30.03^B^ | 9.57 | 28.05^B^ | 9.03 | 32.94^A,C^ | 9.41 | ** |
| Election Affect | 5.45^B^ | 2.23 | 66.53^B^ | 24.59 | 51.35^A,C^ | 25.18 | ** |
| Personal Future | 4.98^B^ | 2.20 | 68.88 | 23.94 | 48.30^A^ | 23.68 | * |
| Collective Future | 6.44^B^ | 2.27 | 73.61^B^ | 21.70 | 59.72^A,C^ | 25.83 | ** |

*Note:* Baseline participant ratings given on Day 1 survey. *p-value* column indicates all instances in which one-way ANOVAs indicated differences in baseline scores by political orientation. **p* < .01. ***p* < .001. Superscripts indicate results of post-hoc tests. *A*: average score is significantly different from Democrats. *B*: average score is significantly different from Republicans. *C*: average score is significantly different from Independents.

**Participants Who Did not Recall a Dream**

How did participants who did not recall a dream compare to those who dreamed about the election, or those who recalled election-unrelated dreams? We explored this using a series of 3 (no dream/election dream/non-election dream) x 3 (political orientation) ANOVAs, with overnight change in feelings toward the election, personal future, and future of the United States as dependent variables. For all 3 measures, overnight change in feelings among participants who did not recall a dream was numerically intermediate between those who recalled an election-unrelated dream, and those who recalled a dream about the election. There were also significant dream x political orientation interactions for all 3 dependent variables (all *p* < .001). Thus, as in the main text, the relationship between dreaming and participants’ overnight change in feelings depended on political orientation. To avoid duplication of results reported in the main text, only post-hoc comparisons involving the “no dream” group will be reported here.

Democrats who did not recall a dream showed less of a negative shift in election-related affect across the night, compared to those who dreamed about the election (*t*(493) = 2.98, *p* = .003, *d* = 0.26), but did not differ significantly from those who recalled an election-unrelated dream. Democrats who did not recall a dream did not differ on overnight change in feelings toward the future, compared to those who dreamed about the election (*p* > .2 for both personal and collective), but did have a greater negative shift in feelings toward the future than those who recalled election-unrelated dreams (personal: *t*(430) = 2.66, *p* = .016, *d* = 0.26; collective: *t*(430) = 4.69, *p* < .001, *d* = 0.45). Independents who did not recall a dream also showed less negative overnight shift in election-related affect than those who dreamed about the election (*t*(181) = 2.75, *p* = .013, *d* = 0.40), but did not differ significantly from those who recalled an election-unrelated dream. Independents who did not recall a dream had less overnight negative shift in feelings toward personal future than those who dreamed about the election (*t*(159) = 2.84, *p* = .015, *d* = 0.41), although this did not reach significance for the collective future (*t*(186) = 1.78, *p* = .152, *d* = 0.26). Independents who did not recall a dream did not differ on overnight change in feelings toward the future from those who reported election-unrelated dreams (both *p* > .5). Republicans who did not recall a dream did not differ significantly from those who recalled dreams about the election or who those recalled election-unrelated dreams, on any measure (all *p* > .194).

To reiterate, while significance of the pairwise comparisons varies, participants who did not recall a dream were numerically intermediate between those who recalled an election-unrelated dream, and those who recalled a dream about the election, on all measures. Given that many dreams are not recalled, this suggests to us that non-recallers may represent a mix of participants who did and did not dream about the election.

**Effects of Political Orientation Considering all Subcategories**

The following results mirror those reported in the main text, except they use all 7 possible levels of political orientation, rather than only the super-ordinate categories of Democrat/Independent/Republican. The pattern of results is similar to those in the main text. However, some comparisons within individual political subgroups do not reach significance here, perhaps owing to the much smaller sample sizes available when participants are split into 7 categories, rather than 3 categories.

**Overnight Change in Feelings About the Election: All Political Categories**

We examined how dreaming about the election was associated with overnight change in feelings toward it using a 2 (election dream: yes/no) x 7 (political orientation) ANOVA, with overnight change in feelings toward the election as the dependent variable. There was a strong main effect of political orientation (*F*(6, 643) = 80.33, *p* <.001, η²ₚ = .428), in which liberal Democrats showed a strong shift toward more *negative* feelings about the election across the night, more moderate Democrats and independents showed less change, and Republican subcategories showed a shift toward more *positive* feelings about the election across the night (see Table S2 and Figure S1). The main effect of dreaming about the election fell short of statistical significance in this model, but was in the same direction as for the model reported in the main text, with participants who dreamed about the election on average feeling worse about it in the morning, relative to those who did not dream about it (*F*(1, 643) = 3.71, *p* = .055, η²ₚ = .006; Figure S1). The election dream x political orientation interaction effect also did not reach statistical significance in this model (*F*(6, 643) = 1.92, *p* = .075, η²ₚ = .018). Independents felt significantly worse about the election the next morning if they had dreamed about it (*t*(79) = 2.56, *p* = .012, *d* = 0.49; Figure S1), but the effect of dreaming did not reach significance in any other individual political subgroup (*p* > .1 for all other political subcategories).

**Table S2**

*Overnight Affect Change by Political Orientation.*

| Participant Classification | Political Orientation | Election Affect | | Personal Future | | Collective Future | |
| --- | --- | --- | --- | --- | --- | --- | --- |
|  |  | Mean | ±SD | Mean | ±SD | Mean | ±SD |
| All  (N = 931) | Moderate R | 1.83 | ±2.72 | 4.59 | ±23.52 | 18.38 | ±30.69 |
|  | Average R | 2.91 | ±2.63 | 13.46 | ±18.11 | 27.97 | ±29.48 |
|  | Conservative R | 1.84 | ±2.47 | 8.21 | ±25.29 | 23.22 | ±29.70 |
|  | Independent | 0.02 | ±3.09 | -4.46 | ±24.98 | -0.43 | ±31.09 |
|  | Moderate D | -2.24 | ±2.59 | -18.53 | ±27.45 | -21.04 | ±30.74 |
|  | Average D | -3.00 | ±2.19 | -20.29 | ±23.40 | -29.39 | ±23.52 |
|  | Liberal D | -3.61 | ±2.72 | -28.60 | ±32.26 | -34.18 | ±27.20 |
| Recalled Dream  (N = 657) | Moderate R | 1.78 | ±2.83 | 3.68 | ±25.70 | 17.43 | ±32.45 |
|  | Average R | 3.05 | ±2.65 | 12.16 | ±18.30 | 29.16 | ±27.07 |
|  | Conservative R | 1.76 | ±2.42 | 8.93 | ±25.90 | 21.37 | ±29.11 |
|  | Independent | -0.07 | ±3.11 | -5.64 | ±26.42 | -1.09 | ±31.67 |
|  | Moderate D | -2.23 | ±2.68 | -16.41 | ±26.66 | -19.06 | ±30.70 |
|  | Average D | -2.96 | ±2.00 | -19.47 | ±23.52 | -27.71 | ±24.10 |
|  | Liberal D | -3.82 | ±2.75 | -29.25 | ±29.92 | -34.08 | ±27.97 |
| Dreamed about Election  (N = 324) | Moderate R | 1.97 | ±3.19 | 4.39 | ±29.08 | 18.64 | ±33.39 |
|  | Average R | 3.12 | ±2.66 | 10.67 | ±15.69 | 33.33 | ±29.00 |
|  | Conservative R | 1.94 | ±2.66 | 13.41 | ±28.06 | 21.94 | ±33.10 |
|  | Independent | -1.04 | ±3.38 | -12.18 | ±29.43 | -6.67 | ±31.09 |
|  | Moderate D | -2.68 | ±2.70 | -23.41 | ±28.10 | -21.70 | ±33.85 |
|  | Average D | -3.35 | ±1.82 | -24.65 | ±25.62 | -34.91 | ±25.74 |
|  | Liberal D | -4.00 | ±2.73 | -30.38 | ±29.43 | -39.80 | ±28.52 |

*Note*. Values represent the change in rating from the day before to the day after the election. Negative numbers indicate an overnight shift toward more negative affect, and positive numbers indicate an overnight shift toward more positive affect. All = all participants who completed both surveys. Recalled Dream = participants who reported having dreamt. Dreamed about Election = participants who reported having dreamt specifically about the election, in any manner. R = Republican and D = Democrat.

**Figure S1**

*Dreaming and Overnight Change in Feelings About the Election and the Future.*

*Note.* The effect of dreaming on overnight change in feelings depended on political orientation. A) Overnight change in feelings about the election by whether participants dreamed about the election and political orientation. B) Overnight change in feelings about the personal future by whether participants dreamed about the election and political orientation. C) Overnight change in feelings about the collective future by whether participants dreamed about the election and political orientation. Error bars = ± SEM.

***Pre-election baseline***

Before the election, feelings about it did not differ between those who later dreamed about it, and those who did not (*F*(1, 643) = 0.10, *p* = .755, η²ₚ < .001). This did not depend on political orientation (election dream x political orientation interaction: *F*(6, 643) = 0.72, *p* = .631, η²ₚ = .007).

**Overnight Change in Future Thinking: All Political Categories**

We next examined how dreaming about the election was associated with overnight changes in feelings about the personal and collective future, using 2 (election dream: yes/no) x 7 (political orientation) ANOVAs. Political orientation strongly affected overnight change in feelings about both the personal (*F*(6, 643) = 31.34, *p* < .001, η²ₚ = .226) and collective (*F*(6, 643) = 60.12, *p* < .001, η²ₚ = .359) future, following the same graded pattern as for feelings towards the election (Table S2 and Figure S1). There were also significant main effects of dreaming about the election, with those who dreamed about the election showing a stronger negative shift in feelings about the future across the night, compared to those who did not dream about the election (for the personal future: *F*(1, 643) = 4.20, *p* = .041, η²ₚ = .006; collective future: *F*(1, 643) = 4.37, *p* = .037, η²ₚ = .007).

The effect of election dreams on feelings about the future significantly depended on political orientation for the personal future (*F*(6, 643) = 2.79, *p* = .011, η²ₚ = .025), and near-significantly for the collective future (*F*(6, 643) = 1.98, *p* = .066, η²ₚ = .018). Dreaming about the election was significantly associated with overnight change in feelings about the personal future among moderate Democrats (*p* = .018), Independents (*p* = .050) and conservative Republicans (*p* = .023) (Figure S1). Dreaming about the election was significantly associated with overnight change in feelings about the collective future among liberal Democrats (*p* = .001) and average Democrats (*p* = .013), as shown in Figure S1.

***Pre-election baseline***

Before the election, feelings about the future did not differ between those who later dreamed about the election, and those who did not (personal future: *F*(1, 643) = 0.04, *p* = .835, η²ₚ < .001; collective future: *F*(1, 643) = 0.01, *p* = .919, η²ₚ < .001). This did not depend on political orientation (election dream x political orientation interaction for personal future: *F*(6, 643) = 0.95, *p* = .458, η²ₚ = .009; for collective future *F*(6, 643) = 0.29, *p* = .940, η²ₚ = .003).

**Mood as a Predictor of Election Dreams (PANAS)**

Election night mood was analyzed using 2 (election dream: yes/no) x 7 (political orientation) ANOVAs, with pre-election PANAS scores as the DV. The night of the election, negative affect was significantly stronger in participants who later dreamed about the it, compared to those who did not (*F*(1, 643) = 8.88, *p* = .003, η²ₚ = .014). This effect did not reach significance for positive affect (*F*(1, 643) = 2.51, *p* = .114, η²ₚ = .004). Dreaming about the election did not significantly interact with political orientation for either positive (*F*(6, 643) = 0.41, *p* = .876, η²ₚ = .004) or negative affect (*F*(6, 643) = 1.25, *p* = .280, η²ₚ = .012).

We examined change in mood across the night using 2 (election dream: yes/no) x 7 (political orientation) ANOVAs, with PANAS change scores as the dependent variable. Those who dreamed about the election showed a greater increase in negative affect across the night compared to those who did not (main effect of election dreams: *F*(1, 643) = 11.65, *p* < .001, η²ₚ = .018). This effect depended on political orientation for negative affect (*F*(6, 643) = 2.27, *p* = .036, η²ₚ = .021), but the interaction did not reach significance for positive affect (*F*(6, 643) = 1.87, *p* = .083, η²ₚ = .017). Dreaming about the election was significantly associated with an overnight increase negative affect only among independents (*p* = .008). Meanwhile, dreaming about the election was significantly associated with an overnight decrease in positive affect only among liberal (*p* = .049) and moderate (*p* = .042) Democrats.

**Changes in Personal vs. Collective Future Thinking Over Time**

To assess how feelings toward the personal vs. collective future changed across the night, we ran a 2 (time: before/after election) x 2 (domain: personal/collective) x 7 (political orientation) ANOVA. Overall, participants felt more positively about their personal future than about the future of the United States (*F*(1, 650) = 276.21, *p* < .001, η²ₚ = .298, Figure 3). But this was qualified by a three-way interaction between time, domain, and political orientation, indicating that political orientation predicted changes in how people felt about the future across the night (*F*(6, 650) = 10.88, *p* < .001, η²ₚ = .091, Figure 3). Democrats’ feelings toward both the personal and collective future became more negative across the night (main effect of time, all *p’s* < .001; Figure S2). Independents’ feelings toward the personal and collective future did not change significantly across the night (*p* = .200). In contrast, Republicans’ feelings toward the future became significantly more *positive* across the night (main effect of time, all *p’s* < .001; Figure S2), especially feelings toward the *collective* future (time x domain interactions, all *p* < .0001). As a result, on the day after the election, moderate and average Republicans felt similarly toward the personal and collective futures, and conservative Republicans actually felt more positively toward the collective future than toward their personal future (*p* = .007; Figure S2).

**Figure S2**

*Feelings About the Personal and Collective Future Converge Overnight in Republicans.*

*Note*. Republicans’ feelings about the future became more positive across the night, but especially for the *collective* future. This led to a convergence in Republicans’ assessment of their personal and collective futures. Error bars = ± SEM.
